# Great Challenges in Pediatrics

**DOI:** 10.3389/fped.2013.00005

**Published:** 2013-03-07

**Authors:** Antonio Francesco Corno

**Affiliations:** ^1^King Fahad Medical City, Cardiovascular Surgery/Pediatric Cardiac SurgeryRiyadh, Saudi Arabia

“We are the world, we are the children”

Michael J. Jackson, Lionel B. Richie

## Children Undoubtedly Represent the Future of the World

As medical practitioners involved in Pediatrics we all have the responsibility of taking care of children. It is our responsibility to protect them from a variety of dangers, including, but not limited to, interruptions during pregnancy, genetic anomalies, perinatal injuries, congenital defects, malnutrition, environmental diseases, infections, poverty, traumas, violence, and exploitation.

When we are in front of a sick child suffering, we should always ask ourselves: “What would you do if this were your child?”

“*There comes a time when we heed a certain call*

*When the world must come together as one*”

The new open-access journal “*Frontiers in Pediatrics*” will offer the experience and organization of specialized sections to serve all individuals interested in the care and the welfare of pediatric patients, from fetal life, through the perinatal and neonatal period, infancy, childhood, and adolescence, until the transitional age to adults.

Endless problems faced by sick children are still waiting for a solution, despite the most recent acquisitions:

a) *Genetic disorders*. The recent decoding and sequencing of the human genome has expanded the horizon of possibilities in the diagnosis of genetic disorders. Researchers and scientists are now facing the difficulties of identifying strengths and limitations of the genome versus exome sequencing to identify the genetic causes of primary immunodeficiencies, before making the information available for potential clinical applications (1).
b) *Pre-natal diagnosis*. Substantial progress has been made in the pre-natal epidemiology in order to identify the congenital heart malformations and facilitate the appropriate treatment as early as possible (2).
  The traditional focus of neonatal screening for inherited metabolic diseases, which is responsible for significant morbidity and mortality unless treatment is initiated early, is also moving toward a genetic and mutational scan across the whole fetal genome in a non-invasive manner by analyzing cell-free fetal DNA in the maternal blood as early as the 5th week of gestational age (3).
  Epidemiological, clinical, cellular, and molecular evidence suggests that the conditions during fetal life play a critical role in developmental programing. Research on the biological mechanisms of fetal programing attracts interest and investigation, and telomere biology could represent the common underlying mechanism connecting fetal programing and subsequent health or susceptibility to complex disorders (4).
c) *Prematurity*. As a consequence of the improvement of pre-natal screening and diagnosis, the recognition of high-risk neonates allowed the referral for delivery in proximity of high level Neonatal Intensive Care Units, with substantial benefits for the neonatal outcomes (5).
d) *Neonatal physiology*. Thanks to the introduction of three-dimensional cardiac magnetic resonance with phase-contrast imaging, major progress has been achieved in acquiring information on the neonatal physiology of the circulation, with the great advantage that this investigative technique can be performed in neonates without sedation or anesthesia (6).
e) *Traumas*. Recent studies have shown the unexpected evidence that the burden of permanent disability resulting from traumatic brain injuries among children is primarily accounted for by mild injuries, rather than by severe injuries. As a result, efforts have to be addressed to prevent, not only severe, but also mild injuries to decrease the levels of disability following traumatic brain injuries (7).
f) *Limited resources*. Despite generalized attempts to diffuse globalization, difficulties still exist in providing medical treatment to geographical areas which have difficult access and/or limited resources. This problem has been documented in the diagnosis of posterior urethral valve, where late referral and presentation are associated with high morbidity and mortality rates (8). Even if couples screening and educational programs have effectively decreased the rates of refusal in couples at risk for beta-thalassemia major, most of the couples in certain geographical areas have a beta-thalassemia major child and related socioeconomic problems, as their reasons for refusing pre-natal diagnosis or termination remain a challenge for the healthcare system even in recent years (9). Good results worldwide have been achieved with prevention and treatment of asthma. Nevertheless socioeconomic and structural barriers for care within health services still remain obstacles to provide optimal treatment of asthma for many children (10).
g) *Introducing new devices and new drugs*. The research and development of drugs and devices for pediatric patients is complicated due to small patient populations, characteristics of pediatric physiology and pathophysiology, practical and ethical difficulties in designing pre-clinical and clinical trials. In pre-clinical trials, it is challenging to identify appropriate experimental models, clinically relevant efficacy end points, assessment of risks and benefits, and methods to monitor cardiovascular safety. Ethical concerns in pediatric clinical trials are due to consideration for the families. Due to the limitations of pre-marketing pediatric studies, post-marketing surveillance of both drugs and devices safety is compulsory in the pediatric population. Solutions for these issues require collaboration between academia, industry, and government as well as creativity in designing pediatric studies (11).
h) *Ambulatory monitoring and care*. Since there is an evident trend to develop and manage healthcare services, it is vital to prevent errors in pediatric ambulatory care. The mistakes most frequently reported include failures in medical treatment, communication, monitoring, patient identification, and the laboratory. Ongoing research is aimed at establishing risk factors for these errors, achieving effective interventions to enhance reporting and improve safety, in order to reduce adverse events and near misses (12). The development of wearable technology for bio-signal monitoring has been recently proved in preterm newborn care, validated by an in-hospital pre-clinical test demonstrating efficiency, reliability, and quality (13).
i) *Continuity of treatment from the pediatric age through the transitional age*. As a result of the advances in medical and surgical treatment during the pediatric age, most patients are now expected to live to adulthood, with a significant increase in the population of adults with congenital defects. Consequently, the transition from a pediatric primary care provider to an adult primary care system has become a critical process in health care management plans, addressing the medical, psychosocial, and educational needs of adolescents and young adults with chronic physical and medical conditions. Useful examples derive from studies of transitional care for children with sickle cell disease (14) and congenital heart defects (15).
j) *Introduction of mathematical models, computers, and robots incare and teaching*. Nowadays the mathematical models with computational fluid dynamics have reached a high level of sophistication. Nowadays a three-dimensional computational model, developed by the finite volume method, can be coupled with an identical hydraulic network describing the entire circulatory system based on pre-stage two hemodynamics. This clinically validated multi-scale approach allows prediction of flow dynamics, cardiac output, mixed venous oxygen saturation, and systemic and cerebral oxygen delivery (16). A strategy for reducing medication errors, morbidity, and mortality in children, which have provided good results, is the use of a computerized provider order entry, resulting in elimination of eligibility errors, ensuring completeness in prescribing fields, reduction in transcription errors, and improved prescribing practices through the use of clinical decision support (17). Future exploration of the development and implementation of computer-assisted learning in medical education can enhance the process and outcomes of medical education, despite the remaining issues of costs, optimal design, use, and integration (18). Recently, researchers have extended upper and lower limbs robot-assisted therapy to children with neurologically based movement disorders arising from cerebral palsy and acquired brain injury or stroke. This new application could lead to new potential benefits to the pediatric population, despite the unavoidable challenges and needs for future development (19).
k) *Application of nanotechnologies*. Recent developments of nanotechnology in the field of cardiovascular diseases are emerging as a potential strategy in dealing with the complications and failures of the conventional treatments. Applications of nanotechnology in medicine are already underway, and offer tremendous potential for diagnostics and therapeutic applications. Widely used biocompatible nano-materials and nano-biotechnological tools have been utilized with high efficacy for biomedical application, such as gene therapy, radiological imaging, targeted delivery systems, and vascular implants (20).
l) *Education and training of the care-givers*. The importance of training non-technical skills is becoming increasingly prominent in the field of enhancing the safety of patients. So far a recognized educational model to support the design of patient safety is lacking, even though a number of theories have been suggested to guide educators in future instructional designs. Further research studies are required to explore which specific aspects of interventions are effective and why, and to assess whether such interventions can impact patient outcomes (21).
m) *Influence of the life style of the parents*. Obesity and the associated and related complications such as diabetes, hypertension, cardiovascular, and respiratory diseases represent the highest risk factor for mortality and morbidity. Childhood obesity, a disturbingly growing problem, is directly related to the number of parent stressors. Parent-perceived stress is correlated to children’s fast-food consumption, an important behavioral indicator of obesity risk. Addressing parent stressors and parent-perceived stress is needed in future research in studying the prevention of child obesity (22).
n) *Ethical and legal issues involved in pediatric studies*. Due to the contemporary regulatory framework for pediatric and neonatal research, and common problems in pediatric research oversight, challenges exist in pediatric research ethics, including balancing risk and benefit, informed consent and assent, and clinical equipments. Three areas of pediatric and neonatal research have been recently investigated in this respect: genomic screening, healthy children donating stem cells, and therapeutic hypothermia for neonates with hypoxic–ischemic encephalopathy (23). Even when consenting minors for genetic research and bio-banking involves ethical and social challenges, other researchers proved that ethical, social, or legal issues were not the leading reasons for refusal of consent, demonstrating a high pediatric consent rate (90%) comparable with that of adults (24).
o) *Research tools in child health*. The development and validation of research tools to measure the results of medical and ambulatory care in pediatric patients are progressing. Recent studies have supported the validity of specific questionnaires for assessing the level of youth friendliness of family medicine services for research purposes, though further validations will be required to allow wider use of this tool in the future (25, 26).

When new discoveries are made available by the scientists involved in experimental and clinical research, the unavoidable consequence is to stimulating more questions, more research, in an endless process toward knowledge.

“*We can’t go on pretending day by day*

*That someone, somehow will soon make a change*”

Whatever our field of involvement in the care of sick children, in basic science, experimental or clinical research, diagnostic investigations, medical or surgical treatment, we have a commitment: active participation to challenge the currently available paradigm in order to improve pediatric care.

The new journal “*Frontiers in Pediatrics*,” thanks to an open platform for mutual exchange, will be a terrific tool providing unexpected views and opinions in the on the pediatric field from all individuals and institutions with interest and motivation.

“*We are the world, we are the children*

We are the ones who make a brighter day

*So let’s start giving*”

Nowadays we don’t have any more valid excuses.

The time when scientists, researchers, and care-givers were allowed to remain in their own little kingdom, enjoying the confidence and the power given by their knowledge limited to a specific field, is gone.

Now is the time for all individuals and institutions dedicated to the care of children to start moving forward, independently of their specialized fields of involvement, sharing their knowledge, and joining their efforts toward a common goal.

This is an essential and timely step if we are to effectively address our pediatric health responsibilities.

The aim of “*Frontiers in Pediatrics*” is to become the tool available to make all “pediatricians” really work together and allow us to say “*we’ll make a better day*” for all children.
